# The role of targeted agents in the adjuvant treatment of colon cancer: a meta-analysis of randomized phase III studies and review

**DOI:** 10.18632/oncotarget.16091

**Published:** 2017-03-10

**Authors:** Bum Jun Kim, Jae Ho Jeong, Jung Han Kim, Hyeong Su Kim, Hyun Joo Jang

**Affiliations:** ^1^ Department of Internal Medicine, Division of Hemato-Oncology, Kangnam Sacred Heart Hospital, Hallym University Medical Center, Hallym University College of Medicine, Seoul, Republic of Korea; ^2^ Center for Colorectal Cancer, Research Institute and Hospital, National Cancer Center, Goyang-si, Republic of Korea; ^3^ Department of Internal Medicine, Division of Gastroenterology, Dongtan Sacred Heart Hospital, Hallym University Medical Center, Hallym University College of Medicine, Hwasung, Republic of Korea

**Keywords:** colon cancer, adjuvant treatment, targeted agent, bevacizumab, cetuximab

## Abstract

There has been debate as to whether targeted agents have beneficial effect when added to adjuvant chemotherapy for patient with colon cancer. We conducted this meta-analysis to investigate the role of targeted agents in the adjuvant treatment of colon cancer. We searched PubMed, MEDLINE, EMBASE, and the Cochrane Library databases. We included phase III trials with the data of disease-free survival (DFS) and adverse events (AEs) of adjuvant treatment with targeted agents. From 5 eligible studies, a total of 9,991 patients with resected colon cancer were included in the meta-analysis of hazard ratio (HR) for 3-year DFS and odds ratio (OR) for grade 3 or higher AEs. The addition of targeted agents showed no improvement of 3-year DFS, compared to standard adjuvant chemotherapy alone (HR = 1.04 [95% confidence interval (CI), 0.96–1.13], *P* = 0.31). In the subgroup analysis according to the type of targeted agents, neither bevacizumab (HR = 1.03 [95% CI, 0.88–1.21], *P* = 0.72) nor cetuximab (HR = 1.11 [95% CI, 0.94–1.31], *P* = 0.22) was associated with improvement of DFS. Moreover, targeted agents significantly increased grade 3 or higher AEs (OR = 1.73 [95% CI, 1.21–2.46], *P* = 0.003) and treatment-related death (OR = 2.15 [95% CI, 1.16–3.99], *P* = 0.02). In conclusion, this meta-analysis demonstrates that the addition of targeted agents to standard adjuvant chemotherapy results in no improvement of DFS with increased severe AEs and treatment-related death in patients with resected colon cancer.

## INTRODUCTION

Colon cancer is the third most common cancer worldwide, accounting for more than 1,300,000 new cases annually and its incidence has sharply increased over the past two decades [1, 2]. Approximately 80% of patients with colon cancer have resectable disease at the time of diagnosis [3]. However, 30–50% of patients who undergo potentially curative surgery experience disease recurrence and die of metastatic diseases [4]. The role of adjuvant chemotherapy to reduce the risk of recurrence after resection has been well established in patients with high-risk stage II or stage III colon cancer [5–10].

Until 2004, adjuvant chemotherapy with 5-fluorouracil and leucovorin (5-FU/LV) was the standard regimen for stage III colon cancer, based on the 24% relative reduction of mortality compared with surgery alone [5, 6]. Since the Multicenter International Study of Oxaliplatin/5-Fluorouracil/Leucovorin in the Adjuvant Treatment of Colon Cancer (MOSAIC) in 2004 [7], the addition of oxaliplatin to fluorouracil-based adjuvant chemotherapy has been considered the standard treatment for high-risk stage II and stage III colon cancer [8]. In the MOSAIC study, oxaliplatin in combination with 5-FU/LV (FOLFOX) showed 3-year disease-free survival (DFS) rate of 78.2%, as compared to 72.9% observed with 5-FU/LV regimen (*P* = 0.002), in patients with stage II and stage III colon cancer. Capecitabine, an oral fluoropyrimidine, can be an effective alternative to 5-FU/LV as adjuvant treatment [9, 10].

Vascular endothelial growth factor (VEGF) antibodies and endothelial growth factor receptor (EGFR) antibodies are molecular targeted agents that have anti-tumor activity by inhibiting tumor angiogenesis or blocking cell signaling pathway. In patients with metastatic colorectal cancer, the addition of targeted agents, such as bevacizumab, cetuximab, or panitumumab to standard chemotherapy has broadened treatment options with significantly improved overall survival [11–13].

After the success of targeted agents in combination with standard chemotherapy in metastatic setting, several clinical trials have been conducted to investigate whether this benefit from the addition of targeted agents to chemotherapy would translate into adjuvant setting [14–17]. In addition, large randomized phase III trial evaluating the efficacy of bevacizumab in combination with capecitabine as adjuvant treatment has been published in 2016 [18].

Until now, there has been debate as to whether targeted agents have beneficial effect without increasing severe toxicities when added to adjuvant chemotherapy. We performed this meta-analysis of randomized phase III trials to reveal the role of targeted agents in the adjuvant treatment for patients with colon cancer.

## RESULTS

### Results of search

**Figure 1:**
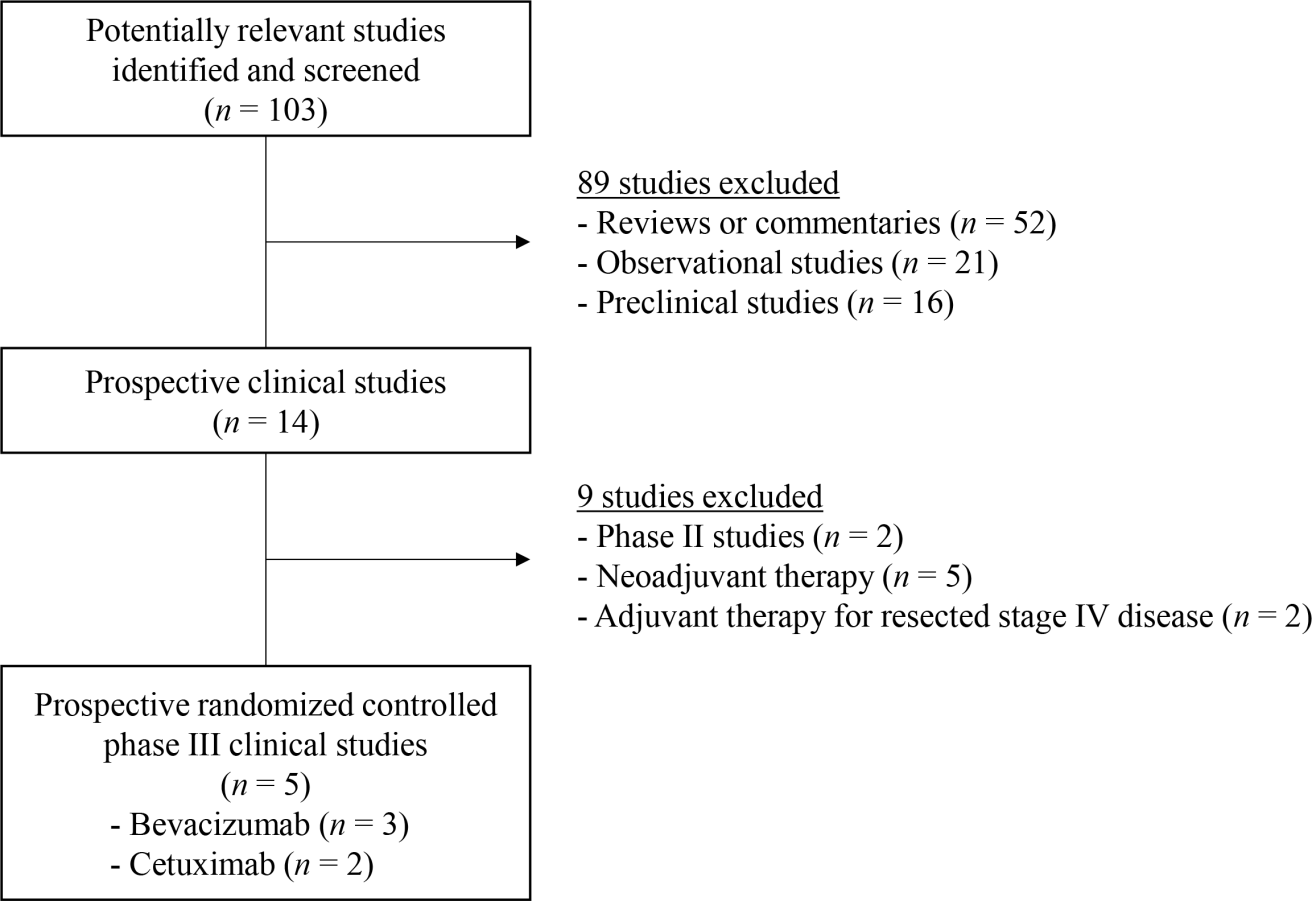
Flow diagram of search process

Figure 1 shows the flowchart of studies through the selection process. A total of 103 potentially relevant studies were identified and screened by searching strategy; 89 were excluded after screening the titles and abstracts. Of the remaining 14 potentially relevant prospective studies, 9 were further excluded by inclusion criteria. Two randomized phase II trials and 5 prospective clinical trials evaluating the adding effect of targeted agents to chemotherapy as neoadjuvant therapy were excluded. Two prospective clinical trials with resected stage IV colorectal cancer patients were also excluded. Finally, 5 randomized controlled phase III clinical trials were included in the meta-analysis [14–18].

### Characteristics of the eligible studies

Table 1 summarizes the characteristics and statistical values of the included studies. In all the studies, the primary endpoint was 3-year DFS. Three studies evaluated bevacizumab-containing adjuvant therapy in patients with stage II or III colon cancer [14, 15, 18], while the remaining 2 studies involved cetuximab in patients with stage III colon cancer [16, 17]. In the two studies with cetuximab, patients had been enrolled regardless of KRAS mutational status. However, 3-year DFS was reported separately according to the KRAS mutational status and we used the results from patients with KRAS wild-type tumor in this meta-analysis.

**Table 1:** Summary of the 5 eligible phase III studies evaluating the role of targeted agents in the adjuvant treatment of colon cancer

Author, trial name (year)	Stage	Treatment arms	No. of patients	3-year DFS rate	HR for 3year-DFS (95% CI)	Incidence of G3/4 AEs	OR for G3/4 AEs (95% CI)	Incidence of TRD	OR for TRD (95% CI)
Allegra *et al*., NSABP C-08 (2011)	II/III	mFOLFOX-6 q2 wks for 6 months + bevacizumab 5 mg/kg q2wks for 12 months	1,334	77.4%	0.89 (0.76–1.04)	77.0%	1.43 (1.20–1.71)	NA	NA
		mFOLFOX6 q2 wks for 6 months	1,338	75.5%		70.0%		NA	
de Gramont *et al*., AVANT (2012)	high risk II /III	FOLFOX-4 q2 wks for 6 months + bevacizumab 5 mg/kg q2 wks for 6 months → 7.5 mg/kg q3 wks for 6 months	960	73%	1.17 (0.98–1.39)	75.9%	1.15 (0.96–1.39)	0.2%	1.97 (0.18–21.74)
		XELOX q3 wks for 6 months + bevacizumab 7.5 mg/kg q3 wks for 12 months	952	75%	1.07 (0.90–1.28)	64.6%		0.4%	
		FOLFOX-4 q2 wks for 6 months	955	76%		73.2%		0.1%	
Kerr *et al*., QUASAR2 (2016)	high risk II /III	Capecitabine alone q3 wks for 6 months + bevacizumab 7.5 mg/kg q3 wks for 12 months	972	75.4%	1.06 (0.89–1.25)	NA	NA	1.6%	1.90 (0.80–4.50)
		Capecitabine alone q3 wks for 6 months	967	78.4%		NA		0.8%	
Alberts *et al*., NCCTG N-0147 (2012)	III	mFOLFOX-6 q2 wks for 6 months + cetuximab 400 mg/m^2^ on C1D1 → 250 mg/m^2^ weekly for 6 months	954	71.5%	1.21 (0.86–1.46)	72.5%	2.40 (2.04–2.83)	0.6%	2.65 (0.70–10.02)
		mFOLFOX-6 q2 wks for 6 months	909	74.6%		52.3%		0.2%	
Taieb *et al*., PETACC-8 (2014)	III	FOLFOX-4 q2 wks for 6 months + cetuximab 400 mg/m^2^ on C1D1 → 250 mg/m^2^ weekly for 6 months	791	75.1%	1.05 (0.85–1.29)	81.9%	2.26 (1.79–2.85)	0.8%	2.41 (0.62–9.34)
		FOLFOX-4 q2 wks for 6 months	811	78.0%		66.7%		0.4%	

AE, adverse event; C1D1, cycle 1 and day 1; CI, confidence interval; DFS, disease free survival; FOLFOX, oxaliplatin with 5-fluorouracil and leucovorin; G3/4, grade 3 or 4; HR, hazard ratio; mFOLFOX, modified FOLFOX; OR, odds ratio; TRD, treatment-related death; NA, not available.

### Three-year disease-free survival

**Figure 2:**
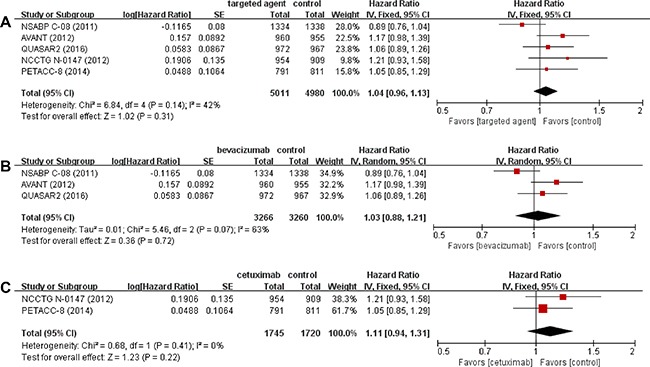
Forest plots of hazard ratios comparing 3-year disease-free survival among all studies (**A**). Subgroup analysis according to the type of targeted agents; bevacizumab (**B**) and cetuximab (**C**).

From the 5 studies [14–18], 9,991 patients were included in the meta-analysis of hazard ratio (HR) for 3-year DFS. We adopted fixed-effect model because there was no significant heterogeneity (*X*^2^ = 6.84, *P* = 0.14, *I*^2^ = 42%). Compared to the standard chemotherapy alone, targeted agents in combination with chemotherapy were not associated with improved 3-year DFS (HR = 1.04 [95% confidence interval (CI), 0.96–1.13], *P* = 0.31) (Figure 2A).

In addition, we performed subgroup analyses according to the type of targeted agents (bevacizumab versus cetuximab), revealing that neither bevacizumab (HR = 1.03 [95% CI, 0.88–1.21], *P* = 0.72) nor cetuximab (HR = 1.11 [95% CI, 0.94–1.31], *P* = 0.22) showed clinical benefit in combination with standard chemotherapy (Figure 2B and 2C).

### Incidence of adverse events

**Figure 3:**
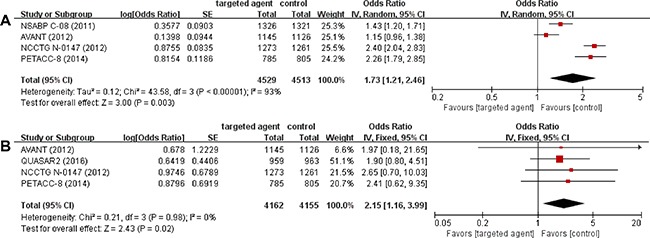
Forest plots of odds ratios comparing the incidence of grade 3 or higher adverse events (**A**) and treatment-related death (**B**).

Four studies [14–17] with 9,042 patients reported the incidence of grade 3 or higher adverse events (AEs) in the intention-to-treat population. We calculated odd ratios (ORs) and their 95% CI from the available data. The random-effect model was used because there was significant heterogeneity (*X*^2^ = 43.58, *P* < 0.00001, *I*^2^ = 93%). The meta-analysis found that adding targeted agents to chemotherapy was associated with 73 % increase of the risk for grade 3 or higher AEs (OR = 1.73 [95% CI, 1.21–2.46], *P* = 0.003) (Figure 3A). The incidence of treatment-related death was reported in 4 studies and was consistently higher in patients who received additional targeted therapy [15–18]. After the meta-analysis, targeted agents were identified to increase significantly the rate of treatment-related death (OR = 2.15 [95% CI, 1.16–3.99], *P* = 0.02) (Figure 3B). The fixed-effect model was adopted because there was no significant heterogeneity (*X*^2^ = 0.21, *P* = 0.98, *I*^2^ = 0%).

## DISCUSSION

We performed this study to investigate the role of targeted agents in patients with resected colon cancer. The meta-analysis of five relevant randomized phase III studies revealed that the addition of bevacizumab or cetuximab to standard adjuvant chemotherapy was not associated with improved DFS and even resulted in worse outcome in terms of toxicity profile.

Several plausible hypotheses may explain the reasons why targeted agents failed to show clinical benefit in the adjuvant treatment setting of colon cancer. Basically, micrometastasis compared to macrometastasis has different pathophysiology and thus the response to targeted agents may be different between adjuvant and metastatic setting. To evolve into metastatic mass from micrometastasis, epithelial-mesenchymal transition and new angiogenesis to supply sufficient blood and make stable metastasis, in which EGFR and VEGF play an important role, are required [19, 20]. Since these essential processes are the biologic targets of cetuximab and bevacizumab, the efficacy of targeted agents may vary depending on the treatment setting. Targeted agents which are cytostatic may have a limited role in micrometastatic disease because, on the basis of Gompertz’s principle [21], micrometastasis tends to grow faster than macrometastasis and is more sensitive to cytotoxic therapy. Micrometastatic disease may develop early resistance to anti-angiogenic therapy by increasing local invasiveness [22] or upregulating pro-angiogenic mechanisms [23].

In addition, adjuvant targeted agents may send tumor cells into dormancy, with re-growth occurring once those agents are discontinued [24]. In the two studies included in this meta-analysis [14, 15], interestingly, the addition of bevacizumab improved DFS temporarily during the early period, but the effect became unfavorable after discontinuation of bevacizumab. Authors described these potentially detrimental outcomes as rebound effects. In several recent studies with preclinical murine models, anti-VEGF therapy resulted in the development of more aggressive disease by tumor hypoxia and inflammatory effects in various tumor types [25–27]. Despite the theoretical concerns about paradoxical effect, however, other clinical studies showed no rebound tumor effects after the withdrawal of VEGR inhibitors [28–30].

In this meta-analysis, the addition of bevacizumab or cetuximab to adjuvant chemotherapy significantly increased grade 3 or higher AEs as well as treatment-related death. Although the incidence of severe AEs was higher in patients receiving additional targeted agents, toxicities did not appear to have decisive influence on the efficacy outcome because no causal relationship between increased toxicities and treatment modification was observed in most studies [14, 15, 17, 18], except one [16]. Major causes of treatment-related death included arterial or venous thromboembolism, gastrointestinal perforation, bleeding, and infection. However, the incidence of treatment-related death was not high enough to affect significantly the final outcome of targeted agents.

As other VEGF or EGFR targeted agents such as panitumumab, aflibercept, and regorafenib come in to use, discovering the predictive biomarkers to identify the correct candidates for targeted agents in adjuvant setting becomes increasingly important. In two studies [14, 18], microsatellite status appeared to act as a predictive marker. Post hoc analysis of the NSABP C-08 trial showed that patients with microsatellite-unstable tumor, not microsatellite-stable tumor, showed a significant survival benefit from the addition of bevacizumab [31]. In QUASAR 2 trial, patients with microsatellite-stable tumor showed significantly worse outcome with the addition of bevacizumab and the influence of microsatellite status was significantly enhanced when analyzed with free CD31 expression level, which is known as the angiogenic marker [18]. Other potential predictive biomarkers including EGFR expression level for cetuximab, KRAS or BRAF mutational status, plasma level of VEGF-A, or VEGF receptors 1 or 2 for bevacizumab have been also investigated but the results were not significant [15, 17, 18].

Of note, our study has several limitations. First, this meta-analysis included the small number of studies currently available. Second, there was significant heterogeneity among studies in the meta-analysis of AEs. We used random-effects model to minimize its influence on the results, but the pooled OR might be affected by the heterogeneity.

In conclusion, this meta-analysis demonstrates that the addition of targeted agents to standard adjuvant chemotherapy results in no improvement of DFS with increased grade 3 or higher AEs and treatment-related death in patients with resected colon cancer. As of now, targeted agents should not be used in the adjuvant treatment of colon cancer. Translational investigations to explore predictive biomarkers are needed to identify the ideal candidates of targeted agents among patients with resected colon cancer.

## MATERIALS AND METHODS

### Searching strategy

We performed a systematic search of PubMed, MEDLINE, EMBASE, and the Cochrane Library databases from January 2000 to January 2017. The following searching terms were used: ‘targeted agent or targeted therapy’, ‘epidermal growth factor receptor inhibitor or EGFR inhibitor’, ‘vascular endothelial growth factor inhibitor or VEGF inhibitor’, ‘cetuximab or bevacizumab’, ‘colon cancer or colon neoplasm or colorectal cancer’, or ‘adjuvant treatment or adjuvant therapy, or adjuvant chemotherapy’. All eligible studies were retrieved and their bibliographies were checked for other relevant publications. When data were unclear or incomplete, the corresponding author was contacted to clarify data extraction.

Eligible studies were required to meet the following inclusion criteria: prospective randomized controlled phase III trials in patients with resected colon cancer; randomization of patients with stage II or III colon cancer to adjuvant treatment with either standard chemotherapy or standard chemotherapy plus targeted agent; providing HR and its 95% CI for DFS; providing OR and its 95% CI for incidence of adverse events.

### Data extraction

The following data were carefully extracted from all eligible studies: first author’s name, year of publication, trial phase, the number of participants, treatment regimens, DFS and its HR with 95% CI, and incidence of grade 3 or higher AEs and their OR with 95% CI.

Data extraction was done independently by two of the authors (BJK and JHJ). If these two authors could not reach a consensus, other authors (JHK and HSK) were consulted to resolve the dispute.

### Statistical analysis

Statistical values used in the analysis were obtained directly from the original article and heterogeneity between studies was estimated using the *I*^2^ inconsistency test and chi-square-based Cochran’s *Q* statistic test in which *P* < 0.1 was taken to indicate the presence of significant heterogeneity. A fixed-effect model (Mantel-Haenszel method) was used to calculate the pooled HR and the pooled OR when substantial heterogeneity was not observed. When substantial heterogeneity was observed, we adopted a random-effects model (DerSimonian-Laird method). Final results were presented with HR or OR and 95% CI. All reported *P*-values were from two-sided versions of the respective test; *P* < 0.05 was considered statistically significant. All meta-analyses and forest plots, annotated with heterogeneity information, were generated using RevMan version 5.2 software.
